# Tissue Regeneration on Rise: Dental Hard Tissue Regeneration and Challenges—A Narrative Review

**DOI:** 10.1155/2024/9990562

**Published:** 2024-04-22

**Authors:** Muhsen Alnasser, Abdullah Hammad Alshammari, Amna Yusuf Siddiqui, Osama Shujaa Alothmani, Rakhi Issrani, Azhar Iqbal, Osama Khattak, Namdeo Prabhu

**Affiliations:** ^1^Department of Restorative Dental Sciences, College of Dentistry, Jouf University, Sakaka, Saudi Arabia; ^2^College of Dentistry, Jouf University, Sakaka, Saudi Arabia; ^3^Department of Endodontics, Faculty of Dentistry, King Abdulaziz University, Jeddah, Saudi Arabia; ^4^Department of Preventive Dentistry, College of Dentistry, Jouf University, Sakaka, Saudi Arabia; ^5^Department of Research Analytics, Saveetha Dental College and Hospitals, Saveetha Institute of Medical and Technical Sciences, Saveetha University, Chennai, India; ^6^Department of Oral and Maxillofacial Surgery and Diagnostic Sciences, College of Dentistry, Jouf University, Sakaka, Saudi Arabia

## Abstract

**Background:**

As people live longer, there is an increasing need for hard tissue regeneration and whole-tooth regeneration. Despite the advancements in the field of medicine, the field of regenerative dentistry is still challenging due to the complexity of dental hard tissues. Cross-disciplinary collaboration among material scientists, cellular biologists, and odontologists aimed at developing strategies and uncovering solutions related to dental tissue regeneration. *Methodology*. A search of the literature was done for pertinent research. Consistent with the Preferred Reporting Items for Systematic Review and Meta-Analysis (PRISMA) 2020 Statement, the electronic databases looked at were PubMed, Science Direct, Scopus, and Google Scholar, with the keyword search “hard dental tissue regeneration.”

**Results:**

Database analysis yielded a total of 476 articles. 222 duplicate articles have been removed in total. Articles that have no connection to the directed regeneration of hard dental tissue were disregarded. The review concluded with the inclusion of four studies that were relevant to our research objective.

**Conclusion:**

Current molecular signaling network investigations and novel viewpoints on cellular heterogeneity have made advancements in understanding of the kinetics of dental hard tissue regeneration possible. Here, we outline the fundamentals of stem hard dental tissue maintenance, regeneration, and repair, as well as recent advancements in the field of hard tissue regeneration. These intriguing findings help establish a framework that will eventually enable basic research findings to be utilized towards oral health-improving medicines.

## 1. Introduction

Hard tissues comprise dental enamel, cementum, and bone [1]. They are also known as calcified tissues because hard tissues include calcium-phosphate minerals [1]. With an aging population worldwide, there is a greater demand than ever for hard tissue regeneration and repair [2, 3]. Malunions, abnormalities, and fractures of the bones have become major global health issues [4, 5]. In 2004, the US gross domestic product was 7.7%, or $849 billion, after missed wages and medical expenses for those with musculoskeletal illnesses [6]. With an aging population, this cost is rising rapidly [6]. Autografts are the best option, but their limited supply and potential for donor site morbidity limit their use. The risk of infection and the increased incidence of nonunion with host tissues present difficulties for allografts. Smart biomaterials, along with tissue engineering structures, present intriguing alternatives to autogenous bone grafts with a plethora of potential applications [3, 7, 8].

The most prevalent disease affecting other hard tissues in humans is tooth caries. Dental caries significantly impairs oral and general health, lowers quality of life, and significantly increases public health expenses [9, 10]. In the US, more than 200 million dental cavity restorations are performed annually, with a $46 billion cost associated with these treatments in 2005 [10]. As life expectancy and the percentage of tooth retention in seniors rise, the need is growing quickly [11]. Mineral loss results from acidogenic bacteria fermenting carbohydrates to create acids, which causes caries [12]. Restorations made to treat dental cavities frequently deteriorate over time, primarily as a result of secondary (recurrent) caries [13]. In addition, more than half of all restorations are replacements for failed restorations [14].

The ongoing increases in tooth loss due to specific bacteria that adhere to teeth metabolize carbohydrates into acid and cause dental caries necessitates the development of novel techniques for tooth regeneration [15]. Conventional dental implants possess a limited lifespan and eventually may be required to be replaced even though metal crowns, porcelain, and resin-based composites have been demonstrated to be quite effective in retaining hard tooth tissues. Under this perspective, there can be significant benefits from developing new techniques for repairing damaged dental hard tissues [15].

The goal of the hard dental tissue regeneration process is to restore the pulp, cement, dentin, and enamel of each individual hard component as well as the entire tooth. Owing to complexity of the dental hard tissues, it becomes challenging to replace a lost tooth with a bioengineered structure that is functional. This complexity and challenge calls for the collaboration of biologic, genetic, or bioengineering approaches [16]. A variety of requirements, including precise dental occlusion, proprioception, appropriate tooth-to-tooth contact, communication of chewing duties, and cosmetic restoration, must be addressed in the construction of the bioengineered teeth [17]. Optimizing the orientation and interactions of the epithelial mesenchymal cell layers with the extracellular matrix is crucial for the creation of teeth with a predetermined form. Creating scaffolds by 3D printing, cell seeding, or other techniques is one strategy to achieve the required distribution of cells inside the matrix [18].

Dentistry and regenerative medicine hold countless assurance for the discipline of dental hard tissue regeneration, which aims to replace or restore lost or damaged dental structures such as cementum, dentin, and enamel [1, 19, 20]. Human teeth are prone to a variety of problems that can cause them to lose their structure and function, such as decay, trauma, and wear and tear. In traditional dental treatments, artificial restorations such as crowns and fillings are frequently placed after diseased tissue has been removed. These methods might not be as functional or long lasting as they could be [1].

The goal of dental hard tissue regeneration is to circumvent these constraints by encouraging the dental structures' organic growth and repair processes. Dental hard tissue regeneration is an intriguing new area in dentistry that may lead to more natural, long-lasting treatments for diseases and damage to the teeth. Better dental treatments and results for patients could result from ongoing study in this area, improving their quality of life and oral health in the process. Hence, the goal for this review is to present a narrative review on dental tissue regeneration.

## 2. Methods

A literature search for relevant studies was completed. The electronic databases searched were PubMed, Science Direct, Scopus, and Google Scholar, utilizing the search keyword “hard dental tissue regeneration” in accordance with the 2020 Statement of Preferred Reporting Items for Systematic Review and Meta-Analysis (PRISMA) [21]. A review of the relevant literature on directed hard dental tissue regeneration was conducted in order to select only those studies that met the review's inclusion criteria. We only took into consideration papers that were published in English. Furthermore, the only materials taken into consideration were original publications, reports, and case series. Textbooks, brief messages, letters, abstracts, interim reports, and book chapters were excluded.

**Figure 1:**
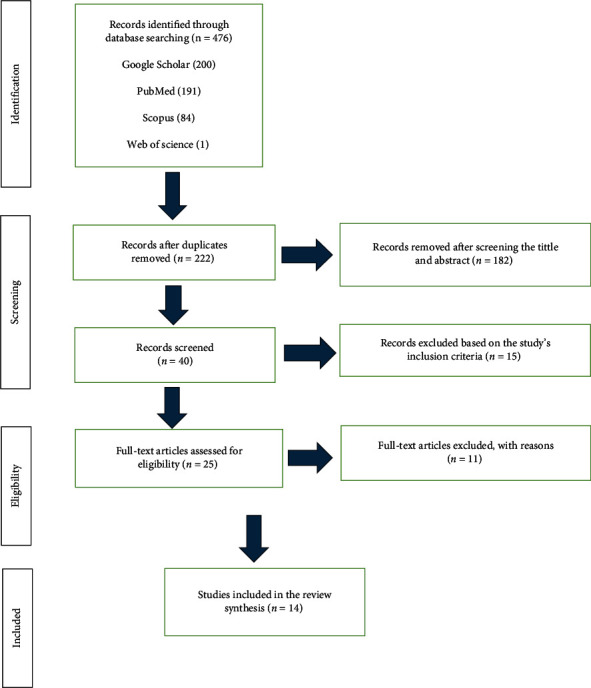
PRISMA flowchart of the study selection process.

Duplicates of every study found using the search procedures were removed after importing them all into an Endnote library. The articles that met the selection criteria were assessed separately by the first and second authors. To settle any disagreements, the third author, who acted as the third reviewer, was consulted. Following this screening, two additional reviewers (the original two authors) examined the chosen records in detail and independently decided whether or not to include them in the review. A third author was again approached in case of any disagreement. The same two reviewers extracted the data into a standard data format. The PRISMA flow diagram shown in Figure 1 was used to present articles that satisfied the eligibility requirements and those that were discarded during the study selection process.

## 3. Results

Using the search method outlined in this study, 476 studies in total were found through database analysis. A total of 222 duplicate articles were removed. Articles without any bearing on guided hard dental tissue regeneration were excluded from consideration. The articles were also excluded for the following reasons: policy statements, thorough reviews, and brief messages. Finally, four studies that were pertinent to our research issue were included in the review.

Despite the fact that there have been a lot of review publications on tooth engineering techniques published recently, we found that only four of them have addressed issues related to dental hard tissue regeneration [1, 22–24] and ten have addressed issues related to hard dental tissues and their genesis [25–34]. These studies provided good descriptions of a number of topics pertaining to dental hard tissue regeneration, yet only one study specifically addressed the connection between the efficacy of the regeneration procedure and the characteristics of biomaterials and/or stem cells. The remaining ten studies illustrate the tooth formation process, which begins during embryogenesis and moves through several stages during pregnancy, childhood, and adolescence until permanent teeth erupt (Table 1). The recent developments in the investigation of many facets and methods of hard dental tissue regeneration are the main topic of this review. The results of the advancement are compared to the problems that this field of study is currently facing.

The included review's findings showcased current developments in hard dental tissue regeneration techniques, highlighted the regeneration of the entire tooth, and described the primary methods for creating scaffolds that are acceptable and offer the right conditions for stem cell development. Stem cells were able to completely contribute to the regeneration of the hard dental tissues and the entire tooth, regardless of the technique used, i.e., scaffold-based or scaffold-free approach [1, 22–24]. Knowledge of the signaling pathways influencing dental tissue genesis could lead to creating innovative cell culture methods and scaffolds with specific functions. Due to the acellular nature of enamel, which makes it impossible to replicate in vitro using a purely cell-based method, functionalized biomaterials are likely to be crucial to the regeneration of hard dental tissues such as dentin and cementum, as well as enamel [25, 27–30, 33]. Researchers have studied and evaluated a great number of potentially suitable biomaterials but only a relatively small number of these have been applied in clinical trials.

Furthermore, review reports and in vitro experiments made up nearly all of the studies contained in this review. Several issues and constraints limit the generalizability of what has been learned about dental tissue regeneration through these investigations. Review reports are vulnerable to bias due to the selection of the included studies. A distorted interpretation of the literature may result from review writers' deliberate or inadvertent favouritism of studies that corroborate their theories or from their disregard of studies with contradicting findings. To examine the impact of different biomaterials or growth factors on dental tissue regeneration, in vitro research frequently uses cell or tissue cultures. These studies might not accurately capture the intricate milieu of the oral cavity while allowing for carefully monitored settings and in-depth molecular investigations. Therefore, research on the regeneration of hard tooth tissue has enhanced our understanding of regenerative therapies, yet there are a number of methodological limitations and issues with generalizability associated with these studies. The discipline must advance, and research findings must be translated into practical, beneficial medical treatments by addressing these limitations through enhanced study designs, standardized methodologies, and rigorous clinical trials.

## 4. Discussion

### 4.1. The Cellular and Molecular Processes Involved in Tooth Development

Developmental and stem cell biologists who research adult tissue regeneration and embryonic morphogenesis have long been interested in teeth because they offer a superb framework for interpreting the molecular principles of organogenesis [30]. In the past few years, molecular signaling networks and new ideas about cellular heterogeneity have helped us learn a lot more about the changing interactions between epithelial and mesenchymal cells that happen during tooth development and homeostasis [30].

The process of developing teeth begins in the embryonic stage and continues in stages during infancy, childhood, and puberty, culminating in the eruption of permanent teeth. The formation of dental tissues is inter-related to one another as one tissue directs the development of the other [25, 27, 35]. Early odontogenesis is characterized by an epithelial-mesenchymal interaction, which is also a model for the formation of other organs such as hair follicles and exocrine glands [31]. The epithelium is derived from the embryonic endoderm, while the mesenchyme is derived from the cranial neural crest. Placodal thickenings of the mouth epithelium along the dental lamina initialize the cellular condensation of the underlying mesenchyme. Subsequently, the tooth primordium undergoes multiple morphological stages, ultimately maturing into a bell stage, bud, and cap. The mesenchyme produces the pulp, periodontal apparatus, and hard materials such as cementum and dentine, whereas the epithelium creates enamel [27].

Subsequently, reciprocal induction begins in the mesenchyme, and epithelial components lose their inductive odontogenic capacity. Numerous molecules interacting in signaling pathways form a signaling program that controls these reciprocal crosstalks. Families that act as morphogenetic inducers, such as Wingless/Int1, Ectodysplasin, Hedgehog, and Bone Morphogenic Proteins are prominent examples of these factors [26, 28, 31]. Signaling centers, which also regulate morphogenesis and coordinate tissue connections, are in charge of determining the size of a single tooth. Cellular signaling also affects tooth shape in addition to tissue variables such as mesenchymal condensation, epithelial contraction, and bone biomechanics [31, 32].

Several recognized stem cell niches exist throughout the development of teeth. Epithelial stem cells reside in the apical end of the advancing epithelium, known as the cervical loop. These cells remain active until the tooth root begins to form [22]. Stem cells aid in the continuous growth of teeth, such as the incisors of mice. The double-layered epithelial root sheath of the hertwig, which elongates the cervical loop, functions as a signaling hub for the formation of tooth roots. It is important to remember that the interaction between the growing alveolar bone and tooth production dictates how teeth form, and this should be considered when constructing whole tooth regeneration techniques [31, 33].

Comprising nonvascularized hard tissues such as dental pulp, soft, vascularized enamel, and dentin, the adult tooth is a complicated organ. Intimately connected to dentin, the dental pulp contains odontoblasts, pericytes, dental pulp stem cells, and other cellular groupings. Nerves allow the pulp and oral environment to convey sensory information, while blood vessels that pierce the pulp provide nutrition to its resident cells. In cases of severe dental damage (e.g., deep cavities), odontoblasts, their progenitors, and dental pulp stem cells can be isolated from the tooth pulp to promote dentin repair [36]. The periodontal ligament, a sophisticated attachment tissue that binds the tooth to the alveolar jawbone and contains odontogenic stem cells, surrounds the tooth [29, 34].

### 4.2. Tissue Engineering Triad

The multidisciplinary discipline of tissue engineering employs engineering and biological science ideas to create biological replacements that have the potential to enhance, preserve, or repair tissue and organ functioning [37]. The foundation of tissue engineering is the regenerative process of biological tissues using growth agents, scaffolds, and progenitor and stem cells [37, 38]. Scaffolds containing the appropriate cells and signaling molecules must be employed to initiate the development of new dental tissue that can homogenize with the surrounding tissues [39–41].

It has been discovered that stem cells of diverse origins are necessary for tissue regeneration. Stem cells include both adult and embryonic stem cells [42]. The source of immature, undifferentiated cells known as embryonic stem cells is the inner cell mass of blastocysts [43, 44]. These cells are capable of perpetual self-renewal and growth. Adult stem and progenitor cells that are still undifferentiated possess the capacity to develop into distinct tissue types [45]. They protect the tissues they dwell in, including blood, skin, bone, and tooth pulp, from breaking down structurally [46].

Growth factors were suggested to be the third crucial element in the triad of tissue engineering and are considered necessary for the process of regeneration. They interact with the extracellular matrix in the surrounding area after being released from cells, immediately offering themselves to cell surface receptors. When growth factors attach to certain cell-membrane-linked receptors, a number of activities and pathways in tissue engineering are initiated [47–50]. These comprise the following: adhesion, development, proliferation, migration, survival, and differentiation into the target cell type. It has been demonstrated that a protein known as bone morphogenetic protein-2 (BMP-2) can convert dental pulp stem/progenitor cells into odontoblasts [47]. It has also been demonstrated that BMP-4 promotes the differentiation of human embryonic stem cells into dental epithelium, which gives rise to teeth [51]. Transfroming growth factor (TGF) also hastens the mineralization process, which dental pulp stem cells control, in addition to helping odontoblast-like cells proliferate [47]. Generally, angiogenesis in the processes of repair and regeneration, wound healing, and growth are all dependent on these growth factor-mediated cell responses [50].

### 4.3. Tooth Regeneration

The ultimate goal of regenerative dentistry is believed to be the replacement of an organ with a whole tooth. For patients, this course of treatment might be their best chance to restore damaged or missing teeth without having to undergo prosthodontic or implantology procedures that require artificial replacements. A complete tooth could be created using a hybrid technique. For instance, a metal or ceramic implant could be used to join a bioengineered tissue structures like a tooth crown or the periodontal ligament, or a prosthetic crown could be joined with a biologically regenerated tooth root, or “bio-root.” In the next several years, creating a complete tooth (a “bio-tooth”) from scratch utilizing just cells and tissues is most likely the aim. This approach is still challenging inspite of the advancements in the basic and translational research [34, 52–55].

Autogenous dental cells from individuals in need of tooth regeneration would be the best way to generate entire teeth. Various approaches to the application of these cells for whole-tooth bioengineering have been established. Combining adult stem cells with teeth embryonic progenitor cells was one idea. Adult stem cells are to demonstrate dental mesenchymal ability and function as a tooth inducer in association with the mesenchymal cells. Alternatively, they should have odontogenic competence and operate as dental epithelium when combined with mesenchymal cells. Young et al. began culturing cells from unerupted porcine tooth buds as early as 2002 [56]. The aggregates were either implanted or generated in vitro on biodegradable scaffolds. As a result, a simple dental crown made of pulp, dentin, and enamel formed. Subsequently, rat and human cells might be used to create bioengineered tooth-like structures that are comparable to these [57, 58]. Ohazama et al. employed an inductive embryonic dental epithelium, which was first implanted under the renal capsule and subsequently placed into adult jaws in 2004, in conjunction with adult mesenchymal stem cells (MSCs) that were not attached to the teeth [59]. Teeth broke through the surface and developed, including the root. Moreover, there was an increase in bone formation. The production of tooth-bud-like structures in vitro was limited to MSCs generated from adipose tissue [60]. To create a whole tooth outside of an embryo, Angelova et al. combined embryonic mouse tooth mesenchyme with human gingival epithelial cells [61].

### 4.4. Enamel Regeneration

The amelogenesis and structure of dental enamel, the hardest tissue in the body of a human, is a highly organized tissue that covers the outer layer of the tooth crown. This material is unique in its mechanical and structural properties due to its high hydroxyapatite content, the way apatite crystals are arranged into enamel prisms, and the way these prisms line up in a picket-fence pattern within a tissue that is very tough and flexible [62–65]. The inner cells of the enamel organ differentiate from specialized epithelial cells known as ameloblasts, which produce enamel [66]. They exhibit polarization and elongation with a protonated Golgi apparatus and endoplasmic reticulum in order to produce and release enamel proteins and to influx phosphate and calcium ions into the enamel matrix that is forming [67, 68]. The three primary proteins found in growing teeth are enamelin, amelogenin, and ameloblastin. Enamel formation requires the presence of enamel proteins [69]. This list now includes an enzyme known as amelotin and a novel protein known as odontogenic ameloblast associated. These were found in the maturation stage of amelogenesis and in the junctional epithelium [70–73]. Ameloblasts undergo reabsorption of water and degradation of enamel proteins during the mature stage of amelogenesis, following the development of the enamel matrix [67, 68]. Ultimately, the adult enamel turns acellular as it goes through apoptosis. Since enamel is not biomineralized like other hard tissues such as bone and dentin, it cannot repair itself if it is broken [74, 75]. Therefore, cellular remineralization of surface demineralized defects is largely, if not entirely, responsible for the reparative healing of damaged enamel [76].

The goal of this research was to restore enamel flaws resulting from caries, trauma, or other causes by developing artificial materials with a hardness similar to enamel [77]. Regretfully, the majority of materials in use today lack the lost tissues' mechanical, physical, and aesthetic qualities [78]. Enamel tissue engineering is encountering numerous challenges in spite of the pressing need for tooth enamel regeneration [79–81]. Among these difficulties are the complex posttranslational protein changes required for crystal growth and the recapitulation of the unique motions of ameloblasts during the formation of hydroxyapatite crystals into enamel prisms [82, 83]. After all these studies and findings, there is still no feasible plan for cell-based in vivo enamel tissue engineering [74]. The main challenge remains producing artificial enamel with the right anatomy to replace lost enamel-forming cells and replicate the prismatic and interprismatic features found in natural enamel [84].

### 4.5. Formation and Regeneration of Dentin

Treatment regarding dentin-pulp complex is most frequently associated with dentin regeneration. It is highly desirable to find ways to preserve pulp vitality because it is necessary for tooth stability and homeostasis. The primary treatment for preserving pulp vitality at the moment is pulp capping; however, this commonly comes with permanent pulp inflammation and reinfections [85]. Therefore, it would be ideal to use novel techniques and biomaterials for the pulp-dentin compound regeneration.

In conventional endodontic therapy, hard tissue development at the apex is created by cleaning and sealing the pulp gap with calcium hydroxide. This formation of the hard tissue creates a barrier for a root-filling substance. This is done by apexification. Because this process does not encourage more root formation, the weak and fragile root canal walls persist, making teeth more vulnerable to subsequent problems [86]. One regenerative endodontic method being studied to circumvent these limitations is remyelization. In this instance, bleeding is produced in order to seal the dental canal and form an endogenous clot of blood that serves as a support structure for the recruitment of growth factors, matrix proteins, and stem cells. Because of root development, apical closure, and the preservation of the tooth's vitality, this process results in the regeneration of the pulp-dentin complex [87, 88]. But because mesenchymal stem cells were present in the blood that was invading, the resultant tissue is more bone-like and associated with connective tissue than the planned pulp-dentin complex [89].

The use of biological printing along with dental progenitor and stem cells employing clinical methods of 3D biofabrication and restoration of oral tissues is now recommended as an alternative to standard dental restorations. It was possible to construct scaffolds with precise, repeatable microarchitectures by using bioinks. These unique dentin-derived extracellular matrix hybrid cell-laden hydrogel bioinks, which were synthesized from alginate and dentin matrix proteins, were examined and showed excellent printability and cell survival at different doses. In addition, the hybrid hydrogels showed that they could be embedded with dentin molecules that are soluble in acid, which improved stem/progenitor cells from apical papilla's odontogenic differentiation and efficiently engineered the pulp-dentin complex [90].

### 4.6. Cementum Formation and Regeneration

The periodontal complex is composed of the alveolar bone, cementum, gingiva, and periodontal ligament. Cementum regeneration occurs directly as a result of treating this compound. Scaffolds used in periodontal complex regrowth not only provide the injured tissue with the support it needs but also frequently serve as a vehicle for bioactive substances like proteins, growth factors, or gene vectors that promote the process of healing and attract nearby stem cells to become involved and settle in. The goal of creating multicompartment scaffolds is to address the various difficulties associated with regenerating several tissues on a single scaffold in periodontal lesions [91]. Furthermore, contemporary research includes cell-based scaffolds, such as cell sheets, in addition to synthetic scaffolds. To use this method, in a lab dish, numerous cell types crucial to periodontal regeneration are cultured until robust cell-cell connections and extracellular matrix synthesis occur. This makes it possible to implant the cell sheet as a substance that resembles a scaffold [92].

The regenerating potential of retro mineral trioxide (MTA), a calcium silicate cement combined with tricalcium phosphate, was examined in a study by Fakheran et al. They found that it produced significantly more new bone and cementum than the control group that was not treated. In addition, the use of biodegradable tricalcium phosphate improves the low rate of biodegradation of MTA [93]. An inorganic calciumphosphate-based scaffold material loaded with BMP-2 was utilized by Wei et al. in a preclinical investigation to treat gum disease in dogs [94]. By itself, the calcium phosphate-based biomaterial significantly increases the regeneration of mineralized tissue and improves the adherence of the teeth to the surrounding tissue as compared to an untreated control and a deproteinized bovinebone mineral utilized as a commercial control. When BMP-2 is added, these promising outcomes could even be increased two- and threefold in terms of the height and area of the remineralized tissues. Unexpectedly, encapsulated BMP-2 had a greater impact on osteogenesis than on oncementogenesis [94]. Wang et al. followed the multicompartment scaffold technique using a bilayered material containing growth factors. The hybrid material consisting of a fibrobblast growth factor-2-loaded propylene-glycol alginate gel (PLGA) on the root surface for ligament repair and a BMP-2-loaded (PLGA/calcium phosphate cement) for periodontal regeneration was evaluated in vivo on nonhuman primates [95]. The authors of a promising rodent study corroborated the findings of the prior examination by demonstrating significantly enhanced cementum and periodontal ligament regeneration in addition to high vascularization of the newly formed periodontal ligament [95, 96].

Cementum has more fluoride than other mineralized tissues, which makes it a great target for catalysis to promote tissue mineralization and speed up cementum regeneration by depositing minerals on the surfaces of tooth roots [97]. In addition, Yang et al. have created collagen scaffolds that include fluorine-containing amorphous calcium phosphates (FACPs) to support the growth of mineralized tissue on tooth-root surfaces that have precise dimensions on a micron scale. The FACP-collagen scaffold was created via the recently created 3D scaffolding method known as Bioskiving, which makes use of decellularized tendon tissues. This made it possible to create hierarchical structures that resemble cementum but with distinctive alternating collagen lamella patterns [37]. Specifically, to arrange the micro- and nanostructures of collagen constructs in three dimensions, the approach can tear away tendon-derived structures (tendon-decellularized structures) and stack sheets of collagen lamellae in alternate orientations [97, 98].

## 5. Clinical Application of the Study

Despite notable advancements in the regeneration of hard dental tissues, there remain several fundamental challenges in implementing the notion of the “processed tooth” in clinical practice. Designing the architecture of bioengineered teeth will require taking into account several crucial factors to achieve this challenging goal. A study on hard dental tissue regeneration may have clinical applications for the creation and use of innovative regenerative therapies to treat tooth injuries or dental caries. Application of a combination of stem cells, growth factors, and bioactive scaffolds to encourage dentin and enamel regeneration in an effort to repair the tooth's damaged structure and function is gaining alot of attention lately. In general, the clinical use of hard dental tissue regeneration presents opportunities for enhancing future research and interdisciplinary approaches to improve tooth restoration and repair, as well as the overall results of dental treatment.

## 6. Conclusion

The use of biopolymeric substances to isolate and regenerate particular periodontal tissues (alveolar bone, cementum, or periodontal ligaments) for the unique dimensions of spatial boundaries has been the focus of numerous attempts in periodontal dental tissue engineering to date. The emergence of mineralized tissue might be particularly promoted by the biopolymer process outlined in this review by means of spatiotemporal control of tissue development towards periodontal issues employing their structures, biological substance immobilization, which lose controls by modification of chemicals, and manufacturing methods for 3D design. By gaining an understanding of signaling pathways influencing the genesis of dental tissue, new approaches to cell culture and the creation of scaffolds with specific functions could be sparked. Given that enamel is an acellular tissue that cannot be replicated in vitro using a purely cell-based method, functionalized biomaterials are likely to be crucial to other dental tissues and hard dental tissues such as dentin and cementum are growing again. Despite the fact that a number of potentially useful biomaterials have already been examined and evaluated, up until this point, only a few of them have been employed in clinical trials. Future research in stem cell-based techniques will probably focus on biomaterials that enable the release of multiple drugs in a progressive and as-needed approach to tailor the different cascade events that take place amid cementogenesis, dentinogenesis, and enamel formation, respectively.

## Figures and Tables

**Table 1:** Research articles included the review.

Prior work	Study tittle	Teeth involve	Conclusion
Olaru et al. [1]	Hard dental tissue regeneration—approaches and challenges	The whole tooth or only one of its component structures	The regeneration of the hard dental tissues and the entire tooth was entirely facilitated by stem cells regardless of the technique used—scaffold based or scaffold free
			The significant amount of differentiation and interconnectedness required for enamel restoration has somewhat hampered the effectiveness of stem cell-based enamel regeneration thus far

Baranova et al. [22]	Tooth formation: are the hardest tissues of the human body hard to regenerate?	Dental tissue regeneration and/or whole-tooth regeneration	Scientific investigation at several levels, including the identification of suitable cell sources with tooth-inductive signals, will be necessary to further the regeneration of entire teeth
			Given that enamel is an acellular tissue that cannot be replicated in vitro using a purely cell-based method, functionalized biomaterials are likely to be crucial to other dental tissues, and hard dental tissues such as dentin and cementum are growing again. In addition, methods to improve 3D organogenesis, 3D printing uses, and the right way to use stimulating chemicals and drugs should all be carefully looked into on a translational level

Ahmed et al. [23]	Tissue engineering approaches for enamel, dentin, and pulp regeneration: an update	Enamel, dental pulpal tissues, and the dentin	Promising methods for preserving the health and repairing the integrity of dental tissues include bioprinting and tissue engineering based on stem and progenitor cells
			Creating culture media without serum or animal products to help cells grow and a clear set of globally recognized markers to separate and identify stem and progenitor cells are two more major issues that must be resolved prior to stem and progenitor cell-based transplantation treatments routinely applied in medical facilities

Kim et al. [24]	Tooth-supporting hard tissue regeneration using biopolymeric material fabrication strategies	Alveolar bone to PDL or PDL to cementum	With their biological immobilization, chemical modification-assisted degradation controls, and manufacturing methods for 3D architectures, biopolymer fabrication techniques can make it easier to create mineralized tissue by controlling where and when tissue grows into periodontal defects
			With synthetic Sharpey's fibres serving as the framework for the teeth, neogenic regulation and fibrous tissue calcification in that area are important for improving biomechanical integration for the hard-to-soft tissue complex and making it easier to restore the regenerated periodontal compound

Tompkins et al. [25]	Molecular mechanisms of cytodifferentiation in mammalian tooth development	Bud, cap, bell, and terminal differentiation	Phenotypic proteins, such as amelogenin and dentin matrix protein 2 of both odontoblasts and ameloblasts, may serve as the best approach during cytodifferentiation

Thesleff [26]	From understanding tooth development to the bioengineering of teeth	The whole tooth	A child's tooth production process can take up too many years when it occurs in vivo. Growth-stimulatory signaling molecules could resolve this issue. Controlling the size, shape, and colour of the dental crown is one of the other issues that still have to be resolved, but these can be resolved using current clinical dentistry techniques. Lastly, there is a significant chance that cells cultured outside of the human body could develop into cancerous cells. The majority of stem cell treatments have this risk, and research is currently being conducted in order to mitigate the chance of cancer

Olley et al. [27]	Expression analysis of candidate genes regulating successional tooth formation in the human embryo	The whole tooth	These results validate the expression of SPROUTY2, GAS1, and RUNX2 throughout the early stages of human tooth formation. While only GAS1 transcripts were discernible in the successional lamina during these early stages of development, the domains of RUNX2 and GAS1 are consistent with a role-influencing function of the primary dental lamina

Huang et al. [28]	Tooth regeneration: insights from tooth development and spatial-temporal control of bioactive drug release	The whole tooth	The intricate spatial-temporal regulatory network of tooth development makes it difficult to identify the critical and necessary components for tooth regeneration. If this research is successful, however, it will liberate restricted parameters that will enable tooth regeneration. Furthermore, before these technologies are clinically translated, additional proof of their in vivo safety is required

Hu et al. [29]	Stem cell-based tooth and periodontal regeneration	Tooth and periodontal	Researchers studied various approaches, such as cell injection, bioroot regeneration, cell-based periodontal regeneration, cell-homing techniques, and epithelial-mesenchymal-based whole tooth regeneration, for tooth and periodontal regeneration. Nonetheless, further research is required to determine the posttransplantation fates and roles of stem cells

Takeo and Tsuji [30]	Organ regeneration based on developmental biology: past and future	The whole tooth	The idea that functional organ regeneration in vivo as a future-oriented regenerative therapy for organ replacement can be accomplished by orthotopically transplanting both an operational organ and a bioengineered organ germ is supported by research

Yu and Klein [31]	Molecular and cellular mechanisms of tooth development, homeostasis, and repair	The whole tooth	Organogenesis, the complex process of tooth formation, involves utilizing multiple pathways that can produce diverse effects in various dental tissue compartments. By using multidisciplinary approaches, we should be able to gain a better understanding of stem cell and dental developmental biology, which will help lay a stronger foundation for future regenerative medicine techniques

Calamari et al. [32]	Tissue mechanical forces and evolutionary developmental changes act through space and time to shape tooth morphology and function	The whole tooth	It is critical to comprehend the processes controlling the acquisition of appropriate tooth morphologies and compositions, as well as the control of progenitor and stem cells throughout tooth growth or regeneration, in order to create stem-cell-based treatments that promote tooth regeneration

Yuan and Chai [33]	Regulatory mechanisms of jawbone and tooth development	Jaw bones and teeth	Shared regulatory systems and other variables tightly connect the development of the jaws and teeth due to their similar cranial neural crest cell origins. In several transgenic mouse models, there is evidence of a close link between the teeth and jaws, where abnormalities are present in both

Hyun et al. [34]	Effect of FGF-2, TGF-*β*-1, and BMPs on teno/ligamentogenesis and osteo/cementogenesis of human periodontal ligament stem cells	Periodontal ligament	According to the findings, fibroblast growth factor-2 mostly causes the stem cells from the human periodontal ligament to differentiate into teno/ligatogenesis and inhibits BMP-2- and BMP-4-induced hard tissue differentiation

## Data Availability

The data used to support the findings of this study are available on request from the corresponding author.
